# Waist Circumference Adjusted for Body Mass Index and Intra-Abdominal Fat Mass

**DOI:** 10.1371/journal.pone.0032213

**Published:** 2012-02-24

**Authors:** Tina Landsvig Berentzen, Lars Ängquist, Anna Kotronen, Ronald Borra, Hannele Yki-Järvinen, Patricia Iozzo, Riitta Parkkola, Pirjo Nuutila, Robert Ross, David B. Allison, Steven B. Heymsfield, Kim Overvad, Thorkild I. A. Sørensen, Marianne Uhre Jakobsen

**Affiliations:** 1 Institute of Preventive Medicine, Copenhagen University Hospital, Copenhagen, Denmark; 2 Division of Diabetes, Department of Medicine, University of Helsinki, Helsinki, Finland; 3 Minerva Medical Research Institute, Helsinki, Finland; 4 Turku PET Centre, University of Turku, Turku, Finland; 5 Departments of Radiology, University of Turku and Turku University Hospital, Turku, Finland; 6 Institute of Clinical Physiology, National Research Council, Pisa, Italy; 7 Departments of Medicine, University of Turku and Turku University Hospital, Turku, Finland; 8 Division of Endocrinology and Metabolism, School of Kinesiology and Health Studies, Department of Medicine, Queen's University, Kingston, Ontario, Canada; 9 Section on Statistical Genetics, Department of Biostatistics, University of Alabama at Birmingham, Birmingham, Alabama, United States; 10 Pennington Biomedical Research Center, Baton Rouge, Louisiana, United States; 11 Department of Epidemiology, School of Public Health, Aarhus University, Aarhus, Denmark; 12 Department of Cardiology, Center for Cardiovascular Research, Aalborg Hospital, Aarhus University Hospital, Aalborg, Denmark; National Institute of Agronomic Research, France

## Abstract

**Background:**

The association between waist circumference (WC) and mortality is particularly strong and direct when adjusted for body mass index (BMI). One conceivable explanation for this association is that WC adjusted for BMI is a better predictor of the presumably most harmful intra-abdominal fat mass (IAFM) than WC alone. We studied the prediction of abdominal subcutaneous fat mass (ASFM) and IAFM by WC alone and by addition of BMI as an explanatory factor.

**Methodology/Principal Findings:**

WC, BMI and magnetic resonance imaging data from 742 men and women who participated in clinical studies in Canada and Finland were pooled. Total adjusted squared multiple correlation coefficients (R^2^) of ASFM and IAFM were calculated from multiple linear regression models with WC and BMI as explanatory variables. Mean BMI and WC of the participants in the pooled sample were 30 kg/m^2^ and 102 cm, respectively. WC explained 29% of the variance in ASFM and 51% of the variance in IAFM. Addition of BMI to WC added 28% to the variance explained in ASFM, but only 1% to the variance explained in IAFM. Results in subgroups stratified by study center, sex, age, obesity level and type 2 diabetes status were not systematically different.

**Conclusion/Significance:**

The prediction of IAFM by WC is not improved by addition of BMI.

## Introduction

Several studies suggest that the association between anthropometric measures of obesity, such as body mass index (BMI) and waist circumference (WC), and mortality is U-shaped [1]–[3]. However, recent large-scale studies have consistently shown that the association between WC and mortality is particularly strong and direct when adjusted for BMI [1], [4]–[8]. The explanation behind this direct association is not established, but one conceivable explanation is that WC adjusted for BMI is a better predictor than WC alone of intra-abdominal fat mass (IAFM), which is presumed to be the most harmful fat depot [9], [10].

We pooled anthropometric and magnetic resonance imaging (MRI) data from European and American samples, and studied the prediction of abdominal subcutaneous fat mass (ASFM) and IAFM by WC alone and by addition of BMI as an explanatory factor.

## Materials and Methods

### Subjects

Subjects (Table S1) were white men and women with no chronic illness, except for type 2 diabetes and a small subset of subjects with stress related angina pectoris symptoms [11]. Subjects were recruited mainly via the general media to participate in clinical studies in Canada [12]–[15] and two sites of Finland; Helsinki [16] and Turku [11], [17]–[20] in the late 1990's and up to 2010. Written informed consent was obtained from each participant in accordance with the local ethical guidelines and with the Helsinki Declaration II.

### Exposure and outcomes

Explanatory variables were BMI (kg/m^2^) and WC (cm). In all centres, height was measured with a height ruler, and body weight was measured with participants wearing light clothes and no shoes. In Canada, WC was measured at the superior edge of the iliac crest or at the level of the lowest rib. In Helsinki, WC was measured midway between spina iliaca superior and the lower rib margin. In Turku, WC was measured at the level of the umbilicus. In all centres, BMI was calculated as weight in kilograms divided by the square of height in meters.

Outcome variables were ASFM and IAFM obtained using MRI. In Canada, abdominal fat mass was determined using 4–5 images acquired from the region extending from 5 cm below to 15 cm above the L4 and L5 intervertebral space using the method described previously [21]. IAFM was defined as intra-peritoneal+retroperitoneal fat mass. In Helsinki, abdominal fat mass was determined by a series of 16 T1-weighted transaxial images acquired from the region extending from 8 cm above to 8 cm below the L4 and L5 intervertebral space using the method described previously [22]. IAFM was defined as intra-peritoneal fat mass. In Turku, abdominal fat mass was determined from a single 10-mm thick axial image at the level of the intervertebral disc L2–L3 using the method described previously [23]. IAFM was defined as intra-peritoneal fat mass, and retroperitoneal fat mass was also assessed. In all centers, an adipose tissue density of 0.9196 g/ml was used to convert the measured volumes into kilos.

Covariates were study centre, sex, age and type 2 diabetes. Type 2 diabetes status was assed from oral glucose tolerance tests or fasting glucose obtained according to standard protocols in the local centers [11]–[20]

### Heterogeneity and pooling of the data

Differences between the study centres, as partly illustrated in Table S1, were addressed by three strategies. First, differences in the measurements of abdominal fat masses were taken into account by converting ASFM and IAFM into centre-specific z-scores. Second, differences in the definitions of IAFM were taken into account by performing the statistical analyses in three different pooled data sets A) pooled data from Canada/Turku using z-scores of IAFM defined as intra-peritoneal+retroperitoneal fat mass, B) pooled data from Helsinki/Turku using z-scores of IAFM defined as intra-peritoneal fat mass, C) pooled data from Canada/Helsinki/Turku using z-scores of IAFM defined as intra-peritoneal+retroperitoneal fat mass in Canada and intra-peritoneal fat mass in Helsinki and Turku. Data from each study centre was also analysed separately using z-scores of the centre specific definitions of IAFM. Third, other differences, e.g. in the measurement site of WC, were taken into account by including centre as a covariate in analyses including all centres.

### Statistical analyses

Analyses were conducted in Stata version 11.2 (Stata Corporation, College Station, Texas; www.stata.com).

The variance explained in ASFM by BMI was calculated as the total adjusted squared multiple correlation coefficient (R^2^) [24] of ASFM obtained from a multiple linear regression model with BMI as explanatory variable. WC was included as an explanatory variable in a second step. Likelihood ratio tests were used to compare the model with BMI with the model with BMI+WC. Similar analyses were conducted for BMI and IAFM, and for WC with BMI added in the second step. Analyses were also conducted with study centre, sex, age and type 2 diabetes included as explanatory factors in a third step. Furthermore, the residuals from each of these models of BMI, WC and their combination were plotted across the distributions of WC and BMI.

To investigate whether the associations between the anthropometric measures and abdominal fat depots were equal across study center, sex, age (cut-off at 50 years), obesity level (cut-off at BMI ≥30 kg/m^2^) and type 2 diabetes status (yes/no), regression analyses were stratified according to each of these factors. Differences between groups were tested by including cross-product terms in the analyses.

Linearity of BMI and WC in the regression analyses was evaluated by restricted cubic splines, and the fit of the models to the data was found acceptable by evaluating the standardized residuals of each model in residual and probit-plots.

## Results

Table 1 provides the basic description of the participants in each of the pooled samples.

**Table 1:** Characteristics of the study participants in each of the samples pooled.

	Canada/Turku (n = 383)	Helsinki/Turku (n = 502)	Canada/Helsinki/Turku (n = 742)
	Median (10–90%-tile)	Median (10–90%-tile)	Median (10–90%-tile)
Age	57 (38; 72)	48 (25.8; 64)	49 (27;68)
Body mass Index (kg/m^2^)	30.6 (26.6; 35.8)	29.7 (23.5; 36.6)	30.2 (24.2; 35.9)
Waist Circumference (cm)	103.8 (91; 115.5)	101 (83.5; 118)	102.3 (86; 117.5)
Abdominal Subcutaneous Fat Mass (kg)	4.6 (2.9; 7.2)	3.9 (1.8; 6.9)	4.2 (2.1; 7.0)
Intra-Abdominal Fat Mass (kg)	3.0 (1.6; 4.8)*	1.5 (0.5; 3.2)#	1.9 (0.6; 4.1)¤
Women in the sample	46.7% (179)	49.8% (250)	50.3% (373)
Subjects with type 2 diabetes	27.1% (104)	36.7% (184)	25.9% (192)

* Intra-Abdominal Fat Mass = intra-peritoneal fat mass+retroperitoneal fat mass.

# Intra-Abdominal Fat Mass = intra-peritoneal fat mass.

¤ Intra-Abdominal Fat Mass = intra-peritoneal fat mass+retroperitoneal fat mass in Canada and intra-peritoneal fat mass in Helsinki and Turku.

Table 2 shows the variance explained in abdominal fat depots by BMI, WC and their combination in each of the pooled samples. The absolute value of R^2^ varied in the samples due to differences in sample characteristics and distribution of the explanatory variables. BMI explained 47%, 65% and 56% of the variance in ASFM, and 11%, 37% and 25% of the variance in IAFM in Canada/Turku, Helsinki/Turku and Canada/Helsinki/Turku, respectively (Table 2, crude models). Addition of WC to BMI added 2%, 1% and 1% to the variance explained in ASFM and 40%, 17% and 27% to the variance explained in IAFM in Canada/Turku, Helsinki/Turku and Canada/Helsinki/Turku, respectively (Table 2, crude models). WC explained 11%, 43% and 29% of the variance in ASFM and 49%, 54%, 51% of the variance in IAFM in Canada/Turku, Helsinki/Turku and Canada/Helsinki/Turku, respectively (Table 2, crude models). Addition of BMI to WC added 38%, 23% and 28% to the variance explained in ASFM and 2%, 0% and 1% to the variance explained in IAFM in Canada/Turku, Helsinki/Turku and Canada/Helsinki/Turku, respectively (Table 2, crude models). Inclusion of study center, sex, age, and type 2 diabetes increased the proportion of variance explained in ASFM and IAFM in all samples (Table 2, adjusted models). As in the crude models, addition of WC to BMI added to the variance explained in IAFM, but only marginally to the variance explained in ASFM. Addition of BMI to WC added to the variance explained in ASFM, but not to the variance explained in IAFM (Table 2, adjusted models). The residuals from the model of BMI, WC and their combination in relation to ASFM and IAFM were similar across the distribution of WC and BMI. So these results were in accordance with the results based on R^2^ (Figure S1 and S2)

**Table 2:** Variance explained in abdominal subcutaneous fat mass and intra-abdominal fat mass by body mass index, waist circumference and their combination in each of the pooled samples.

	Canada+Turku #		Helsinki/Turku ¤		Canada/Helsinki/Turku∥	
	ASFM		ASFM		ASFM	
	Crude	Adjusted*	Crude	Adjusted*	Crude	Adjusted*
	R^2^	R^2^	R^2^	R^2^	R^2^	R^2^
BMI	0.47	0.60	0.65	0.76	0.56	0.70
WC	0.11	0.56	0.43	0.73	0.29	0.66
BMI+WC	0.49	0.62	0.66	0.78	0.57	0.72

	IAFM		IAFM		IAFM	
	Crude	Adjusted*	Crude	Adjusted*	Crude	Adjusted*
	R^2^	R^2^	R^2^	R^2^	R^2^	R^2^
BMI	0.11	0.52	0.37	0.64	0.25	0.58
WC	0.49	0.59	0.54	0.66	0.51	0.63
BMI+WC	0.51	0.59	0.54†	0.67	0.52	0.63

Abbreviations: ASFM, abdominal subcutaneous fat mass. BMI, body mass index- IAFM, intra-abdominal fat mass. R^2^, adjusted squared multiple correlation coefficients. WC, waist circumference.

* Regression models adjusted for study center, sex, age, type 2 diabetes status.

# Intra-abdominal fat mass = intra-peritoneal fat mass+retroperitoneal fat mass.

¤ Intra-abdominal fat mass = intra-peritoneal fat mass.

∥ Intra-abdominal fat mass = intra-peritoneal fat mass+retroperitoneal fat mass in Canada and intra-peritoneal mass in Helsinki and Turku.

p<0.05 for WC and BMI in all models, except for BMI in † where p>0.05.

The results stratified by study center and according to sub-groups of sex, age, obesity level and type 2 diabetes status were not systematically different from the results in the pooled samples (Table S2, S3, S4, S5, S6, S7, S8, S9, S10, S11, S12, S13,S14, crude and adjusted models).

## Discussion

The present study showed, in contrast to the expectation, that the prediction of IAFM by WC was not improved by addition of BMI as an explanatory factor. WC explained a modest proportion of the variation in IAFM, but the proportion was larger than the proportion explained by BMI. Accordingly, the prediction of IAFM by BMI was improved by addition of WC as an explanatory factor. These results were consistent across the different pooled samples and study centers, and in subgroups of sex, age, obesity level and type 2 diabetes status.

Strengths of our study include the use of advanced and precise non-invasive measures of ASFM and IAFM in a large data sample. Abdominal fat masses and WC were measured differently in the study centres, but despite these differences, results were consistent across the study centres. We do therefore not believe that these measurement differences have influenced our results despite some [25], but not other [26] studies suggesting that such measurement differences could have an influence. Due to the large data sample, we could address whether the results differed among sub-groups defined according to sex, age, obesity level and type 2 diabetes status, and results were consistent across these factors. However, limited information on covariates was available, all participants had the same ethnic background, and the majority was overweight and obese. We used R^2^ to assess whether WC adjusted for BMI was a better predictor of IAFM than WC alone. R^2^ is dependent on the distribution of the explanatory variables, and, accordingly, the absolute value of R^2^ varied in the different samples. However, the prediction of IAFM by WC was not improved by addition of BMI as an explanatory factor in any of the samples, which suggests that predictive value of WC and WC adjusted for BMI was not influenced by differences in the distribution of the explanatory variables.

Several large-scale studies have shown that the association between WC and mortality is particularly strong and direct when adjusted for BMI [1], [4]–[8]. One conceivable explanation for this association has been that WC adjusted for BMI is a better predictor of IAFM than WC alone. The variation in WC is believed to originate from variation in ASFM and IAFM, whereas the variation in BMI is believed to originate primarily from variation in subcutaneous fat mass, both at the abdomen and elsewhere. By adjusting WC for BMI, the hypothesis has been that the variation in ASFM is removed from the variation in WC, whereby the variation left in WC adjusted for BMI may directly reflect the variation in IAFM. Our data do not confirm this hypothesis, as addition of BMI to WC did not add to the variance explained in IAFM. Similar to our results, a previous study on white men and women found that addition of BMI to WC added to the variance explained in ASFM, but not to the variance explained in IAFM [27]. The increased mortality risk associated with a high WC in a model adjusted for BMI may, however, not only reflect the effects of high amounts of (intra) abdominal fat mass, but also the effects of low amounts of beneficial body compartments, such as gluteofemoral fat mass or lean body mass [28]–[30]. More studies of WC and WC adjusted for BMI in relation to imaging measurements of fat distribution and body composition are needed to understand the mechanism behind the strong, direct and replicated association between WC adjusted for BMI and mortality [1], [4]–[8].

In conclusion, our results do not support the hypothesis that WC adjusted for BMI is a better predictor of IAFM than WC alone. Therefore, the assumption that WC adjusted for BMI is a better predictor of IAFM than WC alone should be reconsidered.

## Supporting Information

Table S1 **Characteristics of the study participants in each of the included samples.** * Intra-abdominal fat mass = intra-peritoneal+retroperitoneal fat mass. # Intra-abdominal fat mass = intra-peritoneal fat mass.
(DOC)

Table S2 **Variance explained in abdominal subcutaneous fat mass and intra-abdominal fat mass by body mass index, waist circumference and their combination in each study sample.** Abbreviations: ASFM, abdominal subcutaneous fat mass. BMI, body mass index- IAFM, intra-abdominal fat mass. R^2^, adjusted squared multiple correlation coefficients. WC, waist circumference. * Regression models adjusted for sex, age, type 2 diabetes status. p<0.05 for WC and BMI in all models, except for BMI in # and WC in ¤ where p>0.05. ∥Intra-abdominal fat mass = intra-peritoneal fat mass+retroperitoneal fat mass. §Intra-abdominal fat mass = intra-peritoneal fat mass.
(DOC)

Table S3 **Variance explained in abdominal subcutaneous fat mass and intra-abdominal fat mass by body mass index, waist circumference and their combination in the pooled Canada/Helsinki/Turku sample by sex.** Abbreviations: ASFM, abdominal subcutaneous fat mass. BMI, body mass index- IAFM, intra-abdominal fat mass. R^2^, adjusted squared multiple correlation coefficients. WC, waist circumference. * Regression models adjusted for study center, sex, age, type 2 diabetes status. p<0.05 for WC and BMI in all models, except for BMI in # and WC in ¤ where p>0.05. ∥Intra-abdominal fat mass = intra-peritoneal fat mass+retroperitoneal fat mass in Canada and intra-peritoneal mass in Helsinki and Turku.
(DOC)

Table S4 **Variance explained in abdominal subcutaneous fat mass and intra-abdominal fat mass by body mass index, waist circumference and their combination in the pooled Canada/Helsinki/Turku sample by age.** Abbreviations: ASFM, abdominal subcutaneous fat mass. BMI, body mass index- IAFM, intra-abdominal fat mass. R^2^, adjusted squared multiple correlation coefficients. WC, waist circumference. * Regression models adjusted for study center, sex, age, type 2 diabetes status. p<0.05 for WC and BMI in all models, except for BMI in # and WC in ¤ where p>0.05. ∥Intra-abdominal fat mass = intra-peritoneal fat mass+retroperitoneal fat mass in Canada and intra-peritoneal mass in Helsinki and Turku.
(DOC)

Table S5 **Variance explained in abdominal subcutaneous fat mass and intra-abdominal fat mass by body mass index, waist circumference and their combination in the pooled Canada/Helsinki/Turku sample by obesity level.** Abbreviations: ASFM, abdominal subcutaneous fat mass. BMI, body mass index- IAFM, intra-abdominal fat mass. R^2^, adjusted squared multiple correlation coefficients. WC, waist circumference. * Regression models adjusted for study center, sex, age, type 2 diabetes status. p<0.05 for WC and BMI in all models, except for BMI in # and WC in ¤ where p>0.05. ∥Intra-abdominal fat mass = intra-peritoneal fat mass+retroperitoneal fat mass in Canada and intra-peritoneal mass in Helsinki and Turku.
(DOC)

Table S6 **Variance explained in abdominal subcutaneous fat mass and intra-abdominal fat mass by body mass index, waist circumference and their combination in the pooled Canada/Helsinki/Turku sample by type 2 diabetes status.** Abbreviations: ASFM, abdominal subcutaneous fat mass. BMI, body mass index- IAFM, intra-abdominal fat mass. R^2^, adjusted squared multiple correlation coefficients. WC, waist circumference. * Regression models adjusted for study center, sex, age, type 2 diabetes status. p<0.05 for WC and BMI in all models, except for BMI in # and WC in ¤ where p>0.05. ∥Intra-abdominal fat mass = intra-peritoneal fat mass+retroperitoneal fat mass in Canada and intra-peritoneal mass in Helsinki and Turku.
(DOC)

Table S7 **Variance explained in abdominal subcutaneous fat mass and intra-abdominal fat mass by body mass index, waist circumference and their combination in the pooled Canada/Turku sample by sex.** Abbreviations: ASFM, abdominal subcutaneous fat mass. BMI, body mass index- IAFM, intra-abdominal fat mass. R^2^, adjusted squared multiple correlation coefficients. WC, waist circumference. * Regression models adjusted for study center, sex, age, type 2 diabetes status. p<0.05 for WC and BMI in all models, except for BMI in # and WC in ¤ where p>0.05. ∥Intra-abdominal fat mass = intra-peritoneal fat mass+retroperitoneal fat mass.
(DOC)

Table S8 **Variance explained in abdominal subcutaneous fat mass and intra-abdominal fat mass by body mass index, waist circumference and their combination in the pooled Canada/Turku sample by age.** Abbreviations: ASFM, abdominal subcutaneous fat mass. BMI, body mass index- IAFM, intra-abdominal fat mass. R^2^, adjusted squared multiple correlation coefficients. WC, waist circumference. * Regression models adjusted for study center, sex, age, type 2 diabetes status. p<0.05 for WC and BMI in all models, except for BMI in # and WC in ¤ where p>0.05. ∥Intra-abdominal fat mass = intra-peritoneal fat mass+retroperitoneal fat mass.
(DOC)

Table S9 **Variance explained in abdominal subcutaneous fat mass and intra-abdominal fat mass by body mass index, waist circumference and their combination in the pooled Canada/Turku sample by obesity level.** Abbreviations: ASFM, abdominal subcutaneous fat mass. BMI, body mass index- IAFM, intra-abdominal fat mass. R^2^, adjusted squared multiple correlation coefficients. WC, waist circumference. * Regression models adjusted for study center, sex, age, type 2 diabetes status. p<0.05 for WC and BMI in all models, except for BMI in # and WC in ¤ where p>0.05. ∥Intra-abdominal fat mass = intra-peritoneal fat mass+retroperitoneal fat mass.
(DOC)

Table S10 **Variance explained in abdominal subcutaneous fat mass and intra-abdominal fat mass by body mass index, waist circumference and their combination in the pooled Canada/Turku sample by type 2 diabetes status.** Abbreviations: ASFM, abdominal subcutaneous fat mass. BMI, body mass index- IAFM, intra-abdominal fat mass. R^2^, adjusted squared multiple correlation coefficients. WC, waist circumference. * Regression models adjusted for study center, sex, age, type 2 diabetes status. p<0.05 for WC and BMI in all models, except for BMI in # and WC in ¤ where p>0.05. ∥Intra-abdominal fat mass = intra-peritoneal fat mass+retroperitoneal fat mass.
(DOC)

Table S11 **Variance explained in abdominal subcutaneous fat mass and intra-abdominal fat mass by body mass index, waist circumference and their combination in the pooled Helsinki/Turku sample by sex.** Abbreviations: ASFM, abdominal subcutaneous fat mass. BMI, body mass index- IAFM, intra-abdominal fat mass. R^2^, adjusted squared multiple correlation coefficients. WC, waist circumference. * Regression models adjusted for study center, sex, age, type 2 diabetes status. p<0.05 for WC and BMI in all models, except for BMI in # and WC in ¤ where p>0.05. ∥Intra-abdominal fat mass = intra-peritoneal fat mass.
(DOC)

Table S12 **Variance explained in abdominal subcutaneous fat mass and intra-abdominal fat mass by body mass index, waist circumference and their combination in the pooled Helsinki/Turku sample by age.** Abbreviations: ASFM, abdominal subcutaneous fat mass. BMI, body mass index- IAFM, intra-abdominal fat mass. R^2^, adjusted squared multiple correlation coefficients. WC, waist circumference. * Regression models adjusted for study center, sex, age, type 2 diabetes status. p<0.05 for WC and BMI in all models, except for BMI in # and WC in ¤ where p>0.05. ∥Intra-abdominal fat mass = intra-peritoneal fat mass.
(DOC)

Table S13 **Variance explained in abdominal subcutaneous fat mass and intra-abdominal fat mass by body mass index, waist circumference and their combination in the pooled Helsinki/Turku sample by obesity level.** Abbreviations: ASFM, abdominal subcutaneous fat mass. BMI, body mass index- IAFM, intra-abdominal fat mass. R^2^, adjusted squared multiple correlation coefficients. WC, waist circumference. * Regression models adjusted for study center, sex, age, type 2 diabetes status. p<0.05 for WC and BMI in all models, except for BMI in # and WC in ¤ where p>0.05. ∥Intra-abdominal fat mass = intra-peritoneal fat mass.
(DOC)

Table S14 **Variance explained in abdominal subcutaneous fat mass and intra-abdominal fat mass by body mass index, waist circumference and their combination in the pooled Helsinki/Turku sample by type 2 diabetes status.** Abbreviations: ASFM, abdominal subcutaneous fat mass. BMI, body mass index- IAFM, intra-abdominal fat mass. R^2^, adjusted squared multiple correlation coefficients. WC, waist circumference. * Regression models adjusted for study center, sex, age, type 2 diabetes status. p<0.05 for WC and BMI in all models, except for BMI in # and WC in ¤ where p>0.05. ∥Intra-abdominal fat mass = intra-peritoneal fat mass.
(DOC)

Figure S1 **The association between waist circumference, body mass index and the residuals of abdominal subcutaneous fat mass in the pooled Canada/Helsinki/Turku sample.** Abbreviations: ASFM, abdominal subcutaneous fat mass. BMI, body mass index. CAN, Canada. HEL, Helsinki. TUR, Turku. WC, waist circumference. The residuals in the upper panel are derived from a model with WC (left) or BMI (right) as explanatory variables. The residuals in the lower panel are derived from a model with WC and BMI as explanatory variables.
(TIF)

Figure S2 **The association between waist circumference, body mass index and the residuals of intra-abdominal fat mass in the pooled Canada/Helsinki/Turku sample.** Abbreviations: BMI, body mass index. CAN, Canada. HEL, Helsinki. IAFM, intra-abdominal fat mass. TUR, Turku. WC, waist circumference. Intra-abdominal fat mass = intra-peritoneal fat mass+retroperitoneal fat mass in Canada and intra-peritoneal mass in Helsinki and Turku. The residuals in the upper panel are derived from a model with WC (left) or BMI (right) as explanatory variables. The residuals in the lower panel are derived from a model with WC and BMI as explanatory variables.
(TIF)
