# Supplementary material for: Waist Circumference Adjusted for Body Mass Index and Intra-Abdominal Fat Mass
Source: PLoS One. 2012 Feb 24;7(2):e32213. doi: 10.1371/journal.pone.0032213 (PMC3286444; doi:10.1371/journal.pone.0032213)
Supplement: Table S1 — Characteristics of the study participants in each of the included samples. * Intra-abdominal fat mass = intra-peritoneal+retroperitoneal fat mass. # Intra-abdominal fat mass = intra-peritoneal fat mass. (DOC) [file pone.0032213.s001.doc]

|  | **Canada (n=240)** | **Helsinki (n=359)** | **Turku (n=143)** |
| --- | --- | --- | --- |
|  | Median (10-90%-tile) | Median (10-90%-tile) | Median (10-90%-tile) |
| Women in the sample | 51.3% (123) | 54.0% (194) | 39.2% (56) |
| Age | 60.1 (37.0, 72.6) | 42 (24.1, 60.0) | 56 (42.0, 71.0) |
| Body mass Index (kg/m^2^) | 30.9 (26.9, 35.2) | 29.4 (22.8, 36.4) | 29.9 (25.2, 36.6) |
| Waist Circumference (cm) | 104.5 (91.3, 115.4) | 100.0 (81.9, 120.0) | 102.0 (91.0, 116.0) |
| Abdominal Subcutaneous Fat Mass (kg) | 4.7 (3.2, 7.1) | 3.7 (1.7, 6.5) | 4.2 (2.6, 7.8) |
| Intra-Abdominal Fat Mass (kg)* | 3.0 (1.4, 4.9) | - | 2.9 (1.7, 4.6) |
| Intra-Abdominal Fat Mass (kg)# | - | 1.4 (0.4, 3.2) | 1.9 (1.0, 3.2) |
| Subjects with type 2 diabetes | 3.3% (8) | 24.5% (88) | 67.1% (96) |
