# Supplementary material for: Waist Circumference Adjusted for Body Mass Index and Intra-Abdominal Fat Mass
Source: PLoS One. 2012 Feb 24;7(2):e32213. doi: 10.1371/journal.pone.0032213 (PMC3286444; doi:10.1371/journal.pone.0032213)
Supplement: Table S2 — Variance explained in abdominal subcutaneous fat mass and intra-abdominal fat mass by body mass index, waist circumference and their combination in each study sample. Abbreviations: ASFM, abdominal subcutaneous fat mass. BMI, body mass index- IAFM, intra-abdominal fat mass. R2, adjusted squared multiple correlation coefficients. WC, waist circumference. * Regression models adjusted for sex, age, type 2 diabetes status. p<0.05 for WC and BMI in all models, except for BMI in # and WC in ¤ where p>0.05. ∥Intra-abdominal fat mass = intra-peritoneal fat mass+retroperitoneal fat mass. §Intra-abdominal fat mass = intra-peritoneal fat mass. (DOC) [file pone.0032213.s002.doc]

|  | **Canada** | | **Helsinki** | | **Turku** | |  | |
| --- | --- | --- | --- | --- | --- | --- | --- | --- |
|  | **ASFM** | | **ASFM** | | **ASFM** | |  | |
|  | **Crude** | **Adjusted*** | **Crude** | **Adjusted*** | **Crude** | **Adjusted*** |  |  |
|  | **R^2^** | **R^2^** | **R^2^** | **R^2^** | **R^2^** | **R^2^** |  |  |
| BMI | 0.42 | 0.62 | 0.70 | 0.81 | 0.57 | 0.64 |  |  |
| WC | 0.04 | 0.57 | 0.50 | 0.78 | 0.27 | 0.61 |  |  |
| BMI + WC | 0.44 | 0.65 | 0.70 | 0.83¤ | 0.57¤ | 0.65 |  |  |
|  | **IAFM ║** | | **IAFM** § | | **IAFM ║** | | **IAFM** § | |
|  | **Crude** | **Adjusted*** | **Crude** | **Adjusted*** | **Crude** | **Adjusted*** | **Crude** | **Adjusted*** |
|  | **R^2^** | **R^2^** | **R^2^** | **R^2^** | **R^2^** | **R^2^** | **R^2^** | **R^2^** |
| BMI | 0.06 | 0.59 | 0.42 | 0.70 | 0.14 | 0.44 | 0.21 | 0.43 |
| WC | 0.56 | 0.67 | 0.57 | 0.72 | 0.35 | 0.46 | 0.40 | 0.45 |
| BMI + WC | 0.60 | 0.67# | 0.57 | 0.73# | 0.35 | 0.49# | 0.39# | 0.48 |
