# Supplementary material for: Waist Circumference Adjusted for Body Mass Index and Intra-Abdominal Fat Mass
Source: PLoS One. 2012 Feb 24;7(2):e32213. doi: 10.1371/journal.pone.0032213 (PMC3286444; doi:10.1371/journal.pone.0032213)
Supplement: Table S6 — Variance explained in abdominal subcutaneous fat mass and intra-abdominal fat mass by body mass index, waist circumference and their combination in the pooled Canada/Helsinki/Turku sample by type 2 diabetes status. Abbreviations: ASFM, abdominal subcutaneous fat mass. BMI, body mass index- IAFM, intra-abdominal fat mass. R2, adjusted squared multiple correlation coefficients. WC, waist circumference. * Regression models adjusted for study center, sex, age, type 2 diabetes status. p<0.05 for WC and BMI in all models, except for BMI in # and WC in ¤ where p>0.05. ∥Intra-abdominal fat mass = intra-peritoneal fat mass+retroperitoneal fat mass in Canada and intra-peritoneal mass in Helsinki and Turku. (DOC) [file pone.0032213.s006.doc]

|  | **Not Type 2 diabetes** | | **Type 2 diabetes** | |
| --- | --- | --- | --- | --- |
|  | **Crude** | **Adjusted*** | **Crude** | **Adjusted*** |
|  | **R^2^** | **R^2^** | **R^2^** | **R^2^** |
|  | **ASFM** | | **ASFM** | |
| BMI | 0.58 | 0.71 | 0.57 | 0.67 |
| WC | 0.29 | 0.67 | 0.35 | 0.63 |
| BMI + WC | 0.59 | 0.73 | 0.57¤ | 0.68 |
|  | **IAFM** ║ | | **IAFM** ║ | |
| BMI | 0.21 | 0.57 | 0.35 | 0.55 |
| WC | 0.50 | 0.64 | 0.50 | 0.58 |
| BMI + WC | 0.53 | 0.64 | 0.50# | 0.59 |
