# Supplementary material for: Waist Circumference Adjusted for Body Mass Index and Intra-Abdominal Fat Mass
Source: PLoS One. 2012 Feb 24;7(2):e32213. doi: 10.1371/journal.pone.0032213 (PMC3286444; doi:10.1371/journal.pone.0032213)
Supplement: Table S9 — Variance explained in abdominal subcutaneous fat mass and intra-abdominal fat mass by body mass index, waist circumference and their combination in the pooled Canada/Turku sample by obesity level. Abbreviations: ASFM, abdominal subcutaneous fat mass. BMI, body mass index- IAFM, intra-abdominal fat mass. R2, adjusted squared multiple correlation coefficients. WC, waist circumference. * Regression models adjusted for study center, sex, age, type 2 diabetes status. p<0.05 for WC and BMI in all models, except for BMI in # and WC in ¤ where p>0.05. ∥Intra-abdominal fat mass = intra-peritoneal fat mass+retroperitoneal fat mass. (DOC) [file pone.0032213.s009.doc]

|  | **BMI < 30 kg/m^2^** | | **BMI >= 30 kg/m^2^** | |
| --- | --- | --- | --- | --- |
|  | **Crude** | **Adjusted*** | **Crude** | **Adjusted*** |
|  | **R^2^** | **R^2^** | **R^2^** | **R^2^** |
|  | **ASFM** | | **ASFM** | |
| BMI | 0.13 | 0.47 | 0.37 | 0.50 |
| WC | 0.00 | 0.45 | 0.02 | 0.47 |
| BMI + WC | 0.19 | 0.50 | 0.39¤ | 0.54 |
|  | **IAFM** ║ | | **IAFM** ║ | |
| BMI | 0.47 | 0.61 | 0.50 | 0.59 |
| WC | 0.07 | 0.58 | 0.24 | 0.54 |
| BMI + WC | 0.48 | 0.64 | 0.50¤ | 0.60¤ |
