# Supplementary material for: Waist Circumference Adjusted for Body Mass Index and Intra-Abdominal Fat Mass
Source: PLoS One. 2012 Feb 24;7(2):e32213. doi: 10.1371/journal.pone.0032213 (PMC3286444; doi:10.1371/journal.pone.0032213)
Supplement: Table S14 — Variance explained in abdominal subcutaneous fat mass and intra-abdominal fat mass by body mass index, waist circumference and their combination in the pooled Helsinki/Turku sample by type 2 diabetes status. Abbreviations: ASFM, abdominal subcutaneous fat mass. BMI, body mass index- IAFM, intra-abdominal fat mass. R2, adjusted squared multiple correlation coefficients. WC, waist circumference. * Regression models adjusted for study center, sex, age, type 2 diabetes status. p<0.05 for WC and BMI in all models, except for BMI in # and WC in ¤ where p>0.05. ∥Intra-abdominal fat mass = intra-peritoneal fat mass. (DOC) [file pone.0032213.s014.doc]

|  | **Not Type 2 diabetes** | | **Type 2 Diabetes** | |
| --- | --- | --- | --- | --- |
|  | **Crude** | **Adjusted*** | **Crude** | **Adjusted*** |
|  | **R^2^** | **R^2^** | **R^2^** | **R^2^** |
|  | **ASFM** | | **ASFM** | |
| BMI | 0.74 | 0.80 | 0.58 | 0.66 |
| WC | 0.54 | 0.77 | 0.38 | 0.63 |
| BMI + WC | 0.74¤ | 0.82 | 0.58 | 0.68 |
|  | **IAFM** ║ | | **IAFM** ║ | |
| BMI | 0.33 | 0.61 | 0.36 | 0.56 |
| WC | 0.51 | 0.65 | 0.50 | 0.58 |
| BMI + WC | 0.51# | 0.65 | 0.50# | 0.59 |
