# Supplementary figures and images for: Waist Circumference Adjusted for Body Mass Index and Intra-Abdominal Fat Mass
Source: PLoS One. 2012 Feb 24;7(2):e32213. doi: 10.1371/journal.pone.0032213 (PMC3286444; doi:10.1371/journal.pone.0032213)

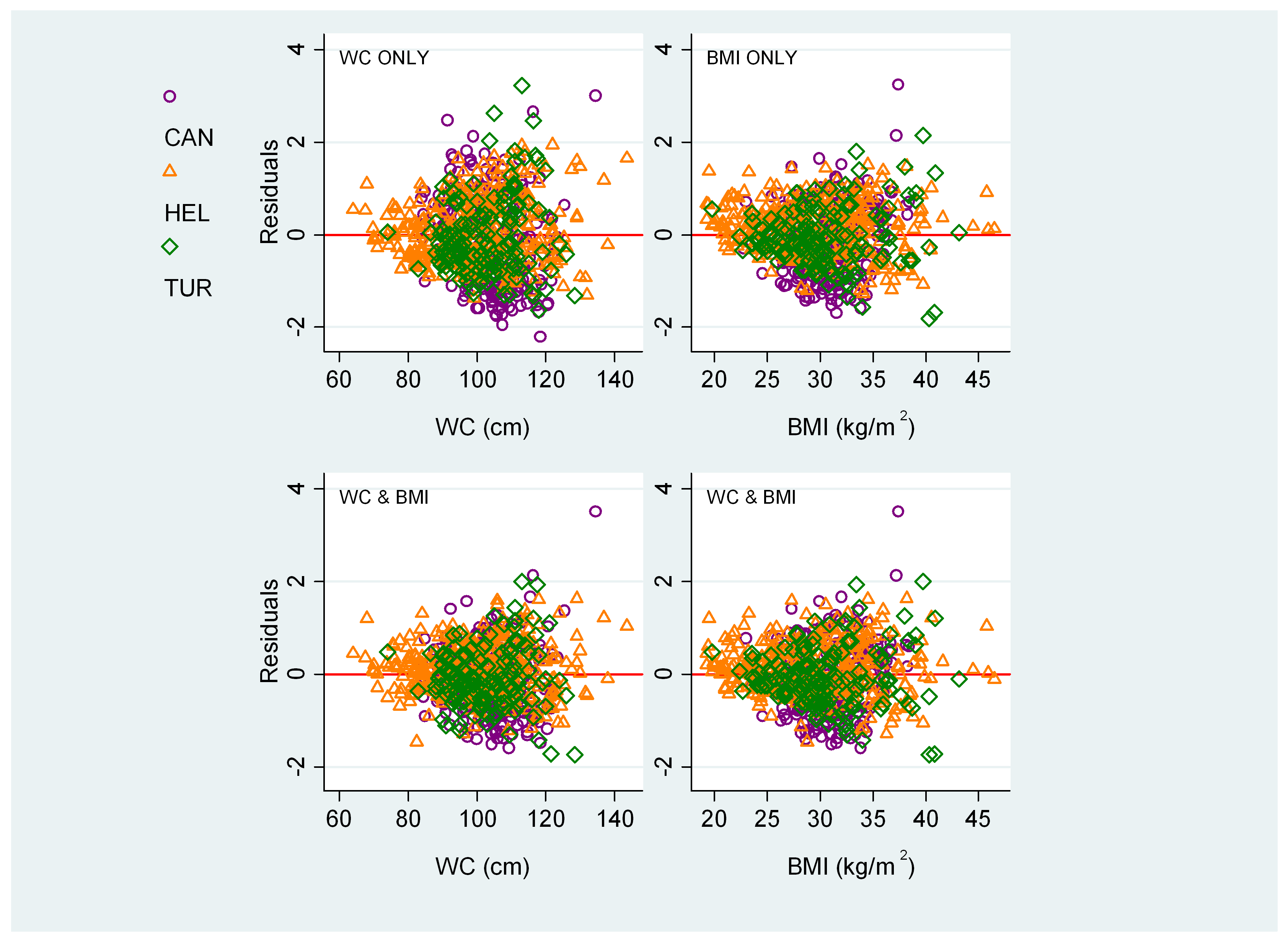

Supplement: Figure S1 — The association between waist circumference, body mass index and the residuals of abdominal subcutaneous fat mass in the pooled Canada/Helsinki/Turku sample. Abbreviations: ASFM, abdominal subcutaneous fat mass. BMI, body mass index. CAN, Canada. HEL, Helsinki. TUR, Turku. WC, waist circumference. The residuals in the upper panel are derived from a model with WC (left) or BMI (right) as explanatory variables. The residuals in the lower panel are derived from a model with WC and BMI as explanatory variables. (TIF) [file pone.0032213.s015.tif]

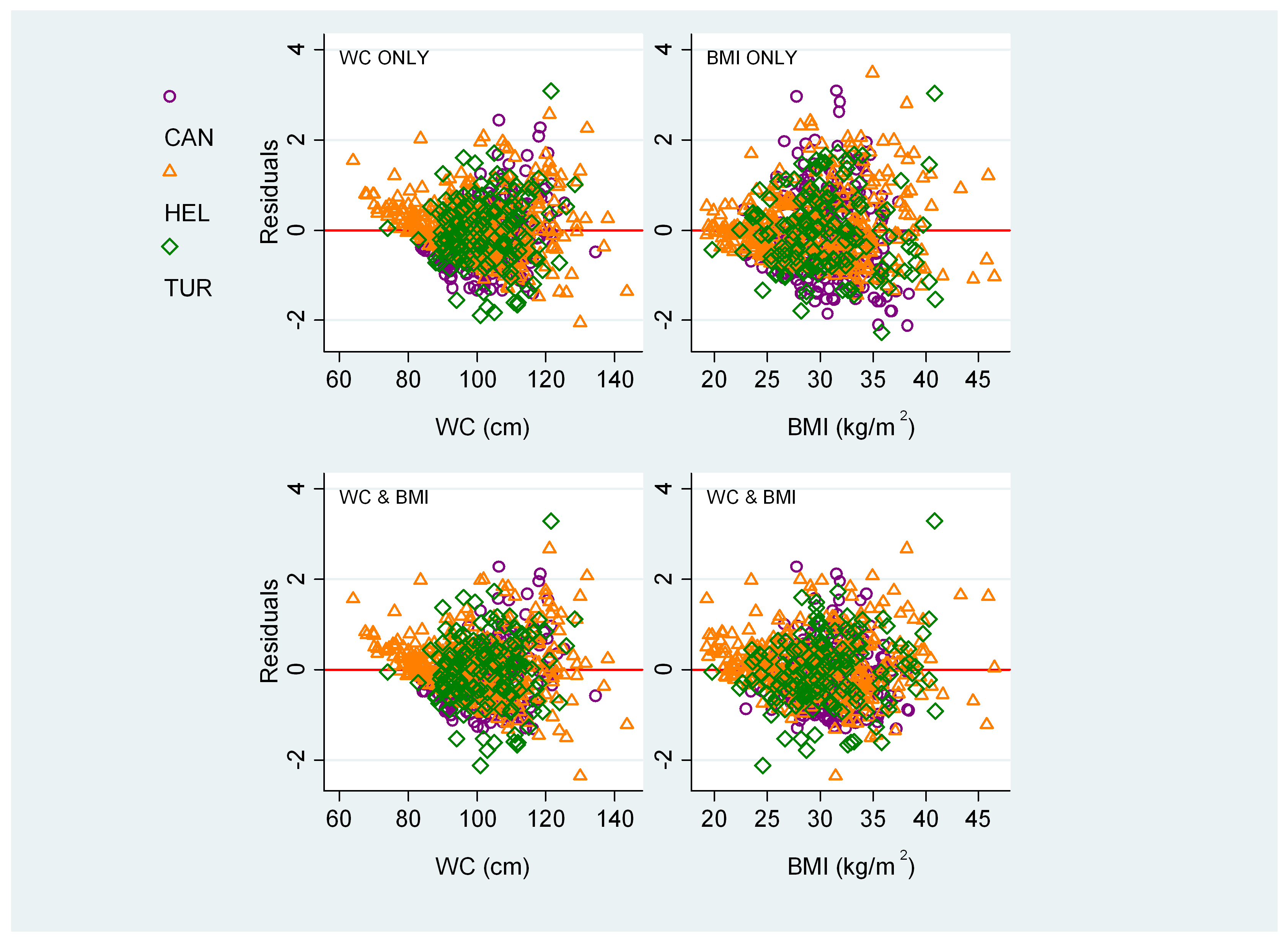

Supplement: Figure S2 — The association between waist circumference, body mass index and the residuals of intra-abdominal fat mass in the pooled Canada/Helsinki/Turku sample. Abbreviations: BMI, body mass index. CAN, Canada. HEL, Helsinki. IAFM, intra-abdominal fat mass. TUR, Turku. WC, waist circumference. Intra-abdominal fat mass = intra-peritoneal fat mass+retroperitoneal fat mass in Canada and intra-peritoneal mass in Helsinki and Turku. The residuals in the upper panel are derived from a model with WC (left) or BMI (right) as explanatory variables. The residuals in the lower panel are derived from a model with WC and BMI as explanatory variables. (TIF) [file pone.0032213.s016.tif]
